# Effects of corrective and breathing exercises on respiratory function of older adults with a history of COVID-19 infection: a randomized controlled trial

**DOI:** 10.1186/s12906-023-04031-7

**Published:** 2023-06-16

**Authors:** Parisa Sedaghati, Korosh Fakhimi Derakhshan, Somayeh Ahmadabadi, Seyed Reza Rahimi Moghaddam

**Affiliations:** 1 Department of Sports Injuries and Corrective Exercise, Faculty of Physical Education and Sport Sciences, University of Guilan, Rasht, Iran; 2 Pediatric Pulmonologist, Pediatric Disease Research Center, Guilan University of Medical Sciences, Rasht, Iran; 3 Department of Physical Education and Sports Sciences, Farhangian University, Tehran, Iran; 4 Council of Strategic Studies and Researches of the General Administration of Sports and Youth of Guilan Province, Rasht, Iran

**Keywords:** Aging, Coronavirus, Posture, Respiratory capacity

## Abstract

**Background:**

Patients with a history of COVID-19 infection may suffer from different physical problems. This study aimed to investigate the effect of corrective and breathing exercises on improving respiratory function among patients with a history of COVID-19 infection.

**Methods:**

In this clinical trial study, thirty elderlies with a history of COVID-19 disease were divided into two groups (mean age 63.60 ± 3.56 experimental, 59.87 ± 2.99 control groups) based on the study inclusion criteria. Exercise interventions included two sections- breathing exercises and corrective exercises in the cervical and thoracic spine. The spirometry test, craniovertebral angle, and thoracic kyphosis test were used. To evaluate differences between variables, paired-samples t-test and ANCOVA were used (*p*-value < 0.01). Also, Eta-squared was measured to assess the effect size.

**Results:**

Results showed a significant difference between the two groups in craniovertebral angle (*P* = 0.001), thoracic kyphosis (*P* = 0.007), and respiratory capacity including Forced expiratory volume in one second (FEV1) (*P* = 0.002), FEV1/FVC (*P* = 0.003), Peripheral oxygen saturation (SPO2) (*P* = 0.001), while no significant differences were observed between two groups in terms of chest anthropometric indices (*P* > 0.01). The Eta-squared value of 0.51 for the Craniovertebral angle and the SPO2 indicates a large effect size.

**Conclusions:**

The results showed the combination of corrective and breathing exercises could improve pulmonary function and correct cervical and thoracic posture in patients with a history of COVID-19 infection. Therefore, corrective and breathing exercises can be helpful as a complementary treatment along with pharmaceutical therapy to reduce chronic pulmonary complications in patients infected with COVID-19.

**Trial registration:**

This research was registered in the Iranian Registry of Clinical Trials (IRCT registration number: IRCT20160815029373N7, First trial registration: 23/08/2021, Registration date: 01/09/2021).

**Supplementary Information:**

The online version contains supplementary material available at 10.1186/s12906-023-04031-7.

## Background

The older people are prone to a high risk of respiratory diseases, such as COVID-19 infection. The Covid-19 pandemic has induced significant physical and mental problems among old people [1].

With aging, physiological changes occur that can affect the performance of various body organs, including the cardiorespiratory and musculoskeletal systems and posture [2]. The disease not only manifests as a pulmonary disease but also is potentially a multifaceted condition that may inflict long-term structural harm to various body organs and the musculoskeletal system [3]. Damage to pulmonary alveoli and, subsequently, severe respiratory system failure in these patients disrupt gas exchange and can lead to death [4, 5]. Various degrees of respiratory dysfunction, physical disability, and mental dysfunction have been reported in elderly patients with COVID-19 [6]. In this disease, the respiratory system is one of the important target tissues, resulting in complications such as dyspnea, decreased lung volume, and hypoxia [3]. Increased respiratory muscle exertion with reduced mobility of the costal and spinal joints are introduced as important factors. So, in patients with asthma, these mechanical changes in the chest wall may lead to soft tissue stiffness and pain [7]. So severe respiratory infections can reduce respiratory function and result in required rehabilitation. Research has been conducted to find effective treatments for COVID-19, and it appears that complementary and alternative medicine may be a viable and valuable option [1]. Interventions improving chest wall flexibility in the thoracic region, such as thoracic mobility exercises, may help address dyspnea while there is little empirical evidence to support these methods [8]. However, little attention has been paid to the effects of Chronic Obstructive Pulmonary Disease (COPD) on musculoskeletal-related variables that may influence the mechanics of breathing, such as posture and spinal motion [9].

Therefore, performing breathing exercises, including those strengthening the respiratory muscles of the chest and boosting lung function (such as deep and calm breathing training, diaphragmatic breathing, and chest exercises) can improve the respiratory condition of affected people and mitigate the stress imposed on the respiratory distress and other related organs [10].

In this regard, Liu et al. (2020) investigated the effect of six weeks of respiratory rehabilitation exercises on the respiratory function, quality of life, mobility, and psychological function of the elderly infected with COVID-19. The results showed significant improvements in FEV1, FEV1/FVC percentage, and the 6-min walking test in the intervention group after six weeks of respiratory rehabilitation [6]. Also, Abodonya et al*.* (2021) investigated the effect of inspiratory muscle exercises on the respiratory function of recovered COVID-19 patients detached from mechanical ventilation and reported significant improvements in FVC% and FEV1% [11]. In addition, the results of a recent study showed that inspiratory muscle training could boost respiratory muscle strength and increase the aerobic capacity of patients with respiratory muscle weakness [12, 13]. Moreover, prior systematic reviews indicate that these exercises can augment the strength and endurance of inspiratory muscles and alleviate dyspnea [14, 15]. In COVID-19 patients, where pulmonary volume and respiratory capacity are reduced, chest exercises (i.e., increasing chest volume) can be of particular importance. These exercises can be performed while lying down, sitting, or standing and in a progressive manner adjusted to patients’ abilities and needs [16].

Clinical evidence has shown that proper respiratory rehabilitation can enhance the prognosis of recovered and discharged COVID-19 patients, maximize the preservation of lung function, and improve their quality of life. Still, there are insufficient studies in the world about the effectiveness of these interventions [6]. Moreover, exercise programs recommended for the rehabilitation of these patients should follow the proper guidelines, including pre-exercise medical examination standards. Exercise protocols should be designed based on the individuals’ characteristics and started immediately after contracting COVID-19, but physical restrictions should be considered an important item in a safe return to exercise [3].

In old people, the high rate of musculoskeletal problems and weak postures such as thoracic kyphosis, forward head, and round shoulder posture, can be associated with physiological complications such as reduced lung volume and respiratory capacity [17]. Some reports demonstrate the high prevalence of these pasture disorders in patients with pulmonary conditions [7]. Also, some studies have shown the positive effects of corrective and breathing exercises on the breathing capacity of people with chronic pulmonary problems [18, 19]. Comprehensive and multifaceted rehabilitation programs under the supervision of specialists have been suggested to be beneficial in the recovery of patients if these interventions are designed based on their respiratory and physical conditions [20]. Therefore, this study aimed to investigate the effects of corrective and breathing exercises on improving the respiratory function of patients with a history of COVID-19.

## Methods

This was a parallel clinical trial. Via different types of announcements to the medical centers across Rasht City, we invited patients between 50–70 years old with a history of COVID-19 disease (six months after recovery from the acute phase of the disease and with a current negative the polymerase chain reaction (PCR) test) to take part in this study. All sample selection, disease diagnosis, and prescription of exercise protocols were performed under the direct supervision of a treatment team, including health specialists and experts. A total of 67 patients volunteered and then, they were evaluated based on the inclusion and exclusion criteria to find the final qualified group. From 67 volunteers, 40 people were eligible based on the inclusion and exclusion criteria. For determining the sample size, according to the study of Liu et al. [6], the means of the FEV1/FVC ratio were regarded as 68.19 ± 6.05 and 61.23 ± 6.43 in the intervention and control groups, respectively.

**Fig. 1:**
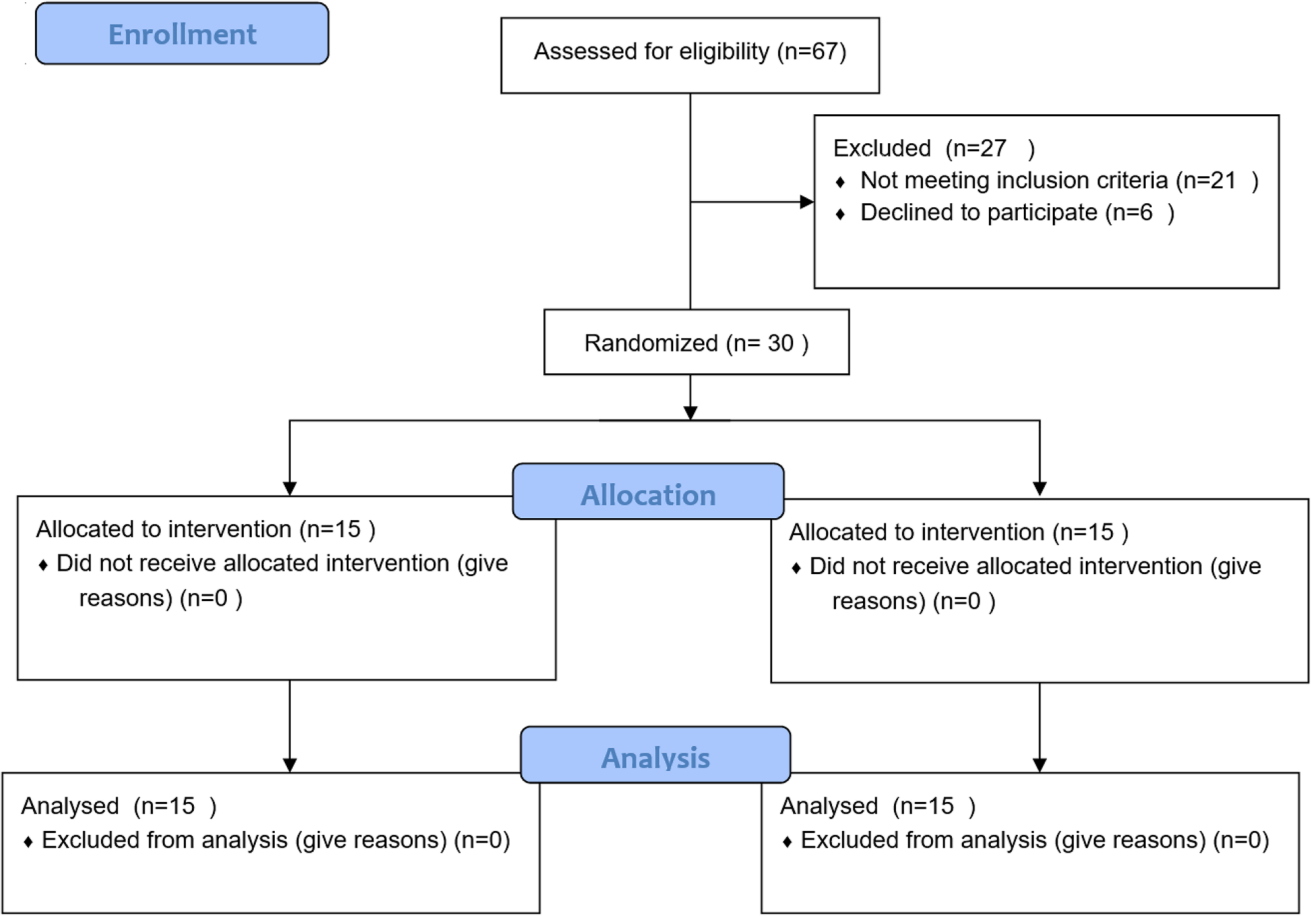
Consort diagram of the study showing sample recruitment and allocation processes

Regarding this continuous outcome variable, the sample size was calculated as 13 subjects per group with an average effect size of 50%, 95% confidence interval, and 80% study power. Due to the possibility of dropouts, the final sample size was determined *n* = 15 for each group (a total of 30 subjects). The sample (*n* = 30) has been selected randomly from the lists of 40 patients who have been qualified. Then, for matching the groups in terms of gender, an equal number of men and women were placed in each group. Figure 1 shows the CONSORT flowchart of the trial and the process of sample recruitment and allocation. Also, reporting checklist for the randomized trial based on the CONSORT guidelines used for this study can be found in Additional file 1. All participants signed informed consent, and all ethical considerations were observed according to the Declaration of Helsinki. This study received an ethics code (IR.KAUMS.REC.1400.021) and a clinical trial registration (IRCT registration number: IRCT20160815029373N7, First trial registration: 23/08/2021, Registration date: 01/09/2021).

### Inclusion criteria

The inclusion criteria consisted of: 1- six months after the definitive diagnosis of COVID-19 and any other acute condition, a current negative PCR test [6], 2- having a non-structural kyphosis abnormality more than 40 degrees [21] or craniovertebral angle less than 51 degrees in a standing position, 3- not having COPD or other acute respiratory diseases, 4- not using aids to walk or perform in daily activities, 5- not having another acute and chronic physical, mental, and psychological disorder (such as cardiovascular, respiratory, cutaneous, arthritis, etc.), 6- no history of neurological diseases such as Parkinson’s and multiple sclerosis, 7- no history of rheumatic or metabolic diseases, 8- the ability to participate in training sessions, 9- not regularly participating in sports activities, 10- age between 50 and 70 years with normal vision, ability to follow simple instructions, and a Mini-Mental State Examination (MMSE) score of higher than 24 [6].

### Exclusion criteria

The exclusion criteria included: 1- the diagnosis of any neurological or orthopedic disease during the study, 2- unwillingness to continue participation in this study, 3- contracting the COVID-19 disease again, 4- the occurrence of any medical problem affecting the safe implementation of the exercise protocol by the patient, 5- the need for starting physiotherapy during the study.

In the experimental group, the subjects and their closest family members were practically trained in the clinic to perform the exercises correctly, including corrective and breathing exercises with supervising and monitoring the health staff. After the health staff ensured exercises were learned correctly by participants, an educational video (a CD) and illustrated instructions were provided to them. Participants' performance was periodically monitored. The experimental group both received their prescription drugs and performed eight weeks of supervised exercise at home (three sessions per week, 30–45 min in each session). The control group received only the necessary treatments prescribed by their physician,

Dependent variables included chest width and depth (using a caliper), craniovertebral angle (using a goniometer), and thoracic kyphosis (using a flexible ruler), and were measured in pre-posttest sessions. Also, a spirometry test was performed to record the FEV1, FVC, SPO2, and FEV1/FVC ratio.

### Thoracic kyphosis assessment

A 60-cm flexible ruler (KERING) measured the kyphosis angle. The ruler was placed between the second and the twelfth dorsal vertebra on the shock appendages of the subject to take the shape of the dorsal vertebra arch. Then, carefully and without changing the ruler’s position, it was placed on white paper to draw the shape of the arcs [22]. The kyphosis angle was then calculated using the formula (ϴ = (4 Arc tan 2 h/1)). A non-structural kyphosis abnormality greater than 40 degrees was the inclusion criterion in this study [21].

### Craniovertebral angle

To check the forward-head posture, the craniovertebral angle was determined by connecting a line from the ear tragus to the spinous appendage of the seventh cervical vertebra and measuring the angle between this line and the horizontal line passing through the spinous appendage. In the present study, a goniometer was used to evaluate this angle. A craniovertebral angle smaller than 51 degrees in the standing position was interpreted as a sign of abnormal forward-head posture, as noted by Kim et al. [23].

### Spirometry testing for evaluating respiratory capacity

The Forced expiratory volume in one second (FEV1), Forced vital capacity (FVC), FEV1/FVC ratio, and peripheral oxygen saturation (SPO2) were measured by spirometry to evaluate the pulmonary function and respiratory capacity. Patients were informed to avoid using medications for 24 h and meals for two hours before the test. The participant was seated and in a resting position half an hour before the test. The spirometry test was performed in the evening (between 4 p.m. and 8 p.m.) by the same expert and in the same place. During the test, the subjects were requested to sit on a chair and put a special nose clip on their nose. After taking a deep breath, a full exhalation was directed into the spirometer tube to record the curve. This procedure was repeated thrice, and the highest values were considered [24]. A Ben caliper was used to measure the depth and width of the chest after the exhalation and in a standing position. A tape measure was used to determine the chest circumference [18].

#### Method of intervention

Exercise interventions included in two parts of corrective exercises—the cervical and thoracic spine- and breathing exercises. Breathing exercises consisted of deep breathing, feather breathing, deep insufflation, deep inhalation and exhalation with concentration, diaphragmatic breathing, and three-stage breathing [25–27].

Corrective exercises related to the cervical and thoracic spine were performed during daily activities such as sitting, standing, and other positions and included myofascial release, stretching, strengthening, and functional exercises [28]. Table 1 provides details of exercises, including sets, repetitions of breathing exercises, and corrective movements adjusted based on the principle of exercise overload after conducting a pilot study.

**Table 1:** Breathing and corrective exercises

Type	Movement content	Duration & intensity	description
Breathing exercises [25–27]	Diaphragmatic breathing	One set/3reps, 60 s rest gradually in two sets	To prevent hyperventilation, only 3 reps performed
	Segmental breathing—deep breathing with emphasis on the expansion of the posterior lower regions—lip bud breathing—Retraining of inspiratory muscles—Lingual pharyngeal breathing—Chest mobility	5 min	
Corrective exercises [28]	chin tuck (sitting and standing), Myofascial release (with foam rolling by the person himself)	One set/5 reps, hold for 10 s, 20-s rest (gradually increase holding to 40 s)	Dorsi latissimus, upper trapezius, erector spine muscles
	Static stretching exercises	One set/4 reps, holding for 20 s, 20-s rest (gradually increase holding to 30 s)	Stretching exercises of the pectoral muscles, latissimus dorsi, sternocleidomastoid, dorsal spine muscles, upper trapezius, and levator scapulae muscles
	Strengthening exercises	Start with a set/10 reps, 60 s rest (gradually increase sets to two)	strengthening exercise of the spinal erector muscle, rhomboid, and middle and lower trapezius
	Functional exercises: dynamic and consistent	Two sets of 8 reps, rest 60 s between sets (gradually increase reps to 10)	combo with neck retraction (T position) on a mat or ball, prone same-side arm leg lift, plank leg raises, and overhead press

During the exercises, subjects were advised to maintain the correct posture that they had previously trained

*reps* Repetitions, *sec* Second

In this research, selected corrective exercises were performed in four steps to reduce kyphosis and open the chest. In the first step, a roller foam was used for the self-myofascial release of the muscles of the dorsal spine, latissimus dorsi, and upper trapezius. The second step included the stretching exercises of the pectoral muscles, latissimus dorsi, sternocleidomastoid, dorsal spine muscles, upper trapezius, and scapulae levator muscles. The third step encompassed the strengthening exercise of the spinal erector muscle, rhomboid, and middle and lower trapezius. In the fourth step, functional exercises included combo with neck retraction (T position) on a mat or ball, prone same-side arm leg lift, plank leg raises, and overhead press on the step [28].

#### Statistical methods

The normality of the data was assessed by the Shapiro–Wilk test. For testing the within-group comparison, paired t-test and for between-group comparison, covariance analysis (ANCOVA) were utilized. *P*-value less than 0.01 to ascertain the statistical significance and eta squared to show the effect size of the intervention on the mean score of variables were performed. The magnitude of d was classified as small (of 0.1 to 0.3), moderate (0.3 to 0.5), or large (0.5 and over) [29]. Data analysis was conducted in SPSS software version 24.

## Results

Table 2 displays the participants’ demographic characteristics, including age, height, weight, body mass index, and waist-to-hip ratio (WHR), as well as baseline values of the research variables. The results of data distribution analysis using the Shapiro–Wilk test confirmed that the data had a normal distribution (*P* ≥ 0.05).

**Table 2:** Means and standard deviations of demographic characteristics and normality distribution

Variable	Experimental Group (Mean ± SD)	Control Group (Mean ± SD))	*P* value
age (y)	63.60 ± 3.56	59.87 ± 2.99	0.011
height (cm)	170.33 ± 8.78	171.47 ± 7.42	0.458
weight (kg)	77.13 ± 14.71	78.60 ± 11.84	0.980
body mass index (kg/m^2^)	26.39 ± 3.17	26.59 ± 2.25	0.034
WHR(cm)	0.96 ± 0.06	0.96 ± 0.07	0.943
Craniovertebral angle (deg)	44.67 ± 0.48	43.47 ± 0.64	0.051
Thoracic kyphosis curve (deg)	45.81 ± 7.10	43.54 ± 5.76	0.385
FVC (lit/sec)	5.63 ± 0.63	5.86 ± 0.49	0.258
FEV1 (lit/sec)	5.09 ± 0.55	5.38 ± 0.44	0.788
FEV1/FVC (%)	90.60 ± 4.09	92.33 ± 4.22	0.163
SPO_2_ (%)	95.33 ± 1.34	95.20 ± 1.82	0.575
chest circumference (cm)	100.73 ± 7.85	99.33 ± 8.30	0.198
chest depth (cm)	21.20 ± 2.00	21.07 ± 1.62	0.127
chest width (cm)	31.00 ± 4.14	30.60 ± 2.29	0.255

*y* Year, *cm* Centimeter, *kg* Kilogram, *deg* Degree, *lit/sec* Liter/second, *%* Percent

The results of variance homogeneity analysis showed that the prerequisite for using ANCOVA (i.e., the homogeneity of variances) was met. Table 3 demonstrates the results of ANCOVA comparing the post-test mean values of the research variables between the two groups after adjustment for pre-test values (as covariates).

**Table 3:** Results of covariance analysis comparing the post-test values between the groups

Variable	Test stage	Mean ± SD	F	*P* value**	Eta squared
Craniovertebral angle(deg)	After experimental	46.07 ± 0.79	27.77	0.001*	0.51
	After control	43.33 ± 0.72			
Thoracic kyphosis curve (deg)	After experimental	44.28 ± 6.51	8.67	0.007*	0.24
	After control	43.68 ± 5.65			
FVC (lit/sec)	After experimental	6.08 ± 0.84	4.036	0.055	0.13
	After control	5.78 ± 0.54			
FEV1 (lit/sec)	After experimental	5.99 ± 0.45	12.15	0.002*	0.31
	After control	5.34 ± 0.67			
FEV1/FVC (%)	After experimental	93.40 ± 3.88	10.80	0.003*	0.28
	After control	92.87 ± 3.80			
SPO2 (%)	After experimental	97.33 ± 1.35	27.76	0.001*	0.51
	After control	95.13 ± 1.40			
chest circumference (cm)	After experimental	102.93 ± 7.23	4.66	0.04	0.147
	After control	99.20 ± 8.27			
chest depth (cm)	After experimental	21.47 ± 2.20	1.39	0.24	0.04
	After control	21.13 ± 1.45			
chest width (cm)	After experimental	31.53 ± 3.60	1.90	0.17	0.06
	After control	30.80 ± 2.17			

*deg* Degree, *lit/sec* Liter/Second, *%* Percent, *cm* Centimeter

^*^Significant at *P* < 0.01

^**^Adjusted based on pre-test values

The results of ANCOVA showed significant differences in postural (i.e., the craniovertebral angle and thoracic kyphosis curve) and respiratory capacity (FEV1, FEV1/FVC, SPO2) variables between the two groups (*P* < 0.01), with no significant differences in chest anthropometric indices (*P* > 0.01). The Eta-squared value for the Craniovertebral angle and the SPO2 was 0.51 indicating a large effect size while the value for the FEV1 was 0.31 indicating a moderate effect size.

The paired t-test was used to check differences between pre-test and post-test values in each group (Table 4). The results of the paired t-test showed that in the experimental group, the exercises significantly affected postural (i.e., craniovertebral angle and thoracic kyphosis curve) and respiratory capacity (FEV1, FEV1/FVC, SPO2) variables in the elderly with a history of COVID-19 infection (*P* < 0.01); however, no significant effect was observed on chest anthropometric parameters (*P* > 0.01). No significant difference was observed in the pre-and post-test values of the research variables in the control group (*P* > 0.01).

**Table 4:** Comparison of pre- and post-test values of variables in each study group

Groups	Experimental group (*N* = 15)		control group (*N* = 15)	
	T	*P* value	T	*P* value
Craniovertebral angle(deg)	-6.54	0.001*	1.46	0.16
Thoracic kyphosis curve(deg)	4.08	0.001*	-0.39	0.69
FVC (lit/sec)	-2.78	0.015	0.52	0.61
FEV1 (lit/sec)	-5.10	0.001*	0.25	0.79
FEV1/FVC(%)	-6.38	0.001*	-1.74	0.10
SPO2(%)	-4.97	0.001*	0.25	0.80
Circumference of the chest(cm)	-1.85	0.08	1.00	0.33
Chest depth(cm)	-2.25	0.04	-0.56	0.58
chest width(cm)	-1.83	0.08	-1.38	0.18

*deg* Degree, *lit/sec* Liter/Second, *%* Percent, *cm* Centimeter

^*^Significant at *P* < 0.01

## Discussion

In our study on the elderly with a history of COVID-19 disease, the results revealed significant differences in postural (i.e., craniovertebral angle and thoracic kyphosis curve) and respiratory capacity (FEV1, FEV1/FVC, SPO2) between the experimental (performing corrective and breathing exercises) and the control group. Also, the Craniovertebral angle and the SPO2 showed a large effect size while the effect size of FEV1 was a moderate level.

In this regard, head and back spine posture and respiratory capacity parameters significantly improved in subjects performing eight weeks of corrective and breathing exercises compared to the control group. In line with these findings, in the study of Gad et al*.* (2014), corrective exercises were used to reduce kyphosis and improve pulmonary function in patients with COPD. In the recent research, the subjects were randomly divided into two groups; one group received a combination of corrective and breathing exercises, and the second group received only breathing exercises, and results showed improvements in pulmonary function in both groups, but the kyphosis angle was reduced only in the group performing corrective exercises [30]. Moreover, in another study, Zarneshan et al*.* (2018) investigated the effectiveness of a 12-week breathing and aerobic exercise in controlling asthma and health improvement among 24 asthmatic women and reported a significant increase in FEV1 and better asthma control in the experimental group [31]. Overall, research evidence suggests that breathing exercises can improve the quality of life in people with respiratory problems by boosting respiratory function. Also, such exercises can reduce the need for using bronchodilators by improving pulmonary function via promoting positive physiological effects on the airways, alleviating the hypersensitivity of the airways, and reducing inflammation [32].

Therefore, prolonged and chronic respiratory diseases, such as asthma, can induce contractile changes or muscle imbalances in the neck and shoulder. Compared to healthy people, patients with mild or severe persistent asthma maintain their head and shoulders more forward and show a lower level in the chest wall, internal shoulder rotation, and spine flexibility [9, 33].

Also, the tendency to adopt a bent posture (head forward and hyper kyphosis) is particularly common in old patients, which leads to undesired structural and functional changes, impaired performance of motor skills, and reduced adaptation of the patient to the environment [34].

Therefore, corrective exercises in combination with posture correction training can reduce these complications and improve the volume, chest expansion, and pulmonary function as well. In line with this result, Jang et al*.* (2015) investigated the status of thoracic hyper kyphosis and bend posture in elderly women and reported eight weeks of chest corrective exercises significantly improved thoracic kyphosis status and bend posture. They reported evidence of these positive changes through kyphosis improvement by 3.45 degrees in the normal position and 3.50 degrees in the bending position after eight weeks of training [35]. In another study, Katzman et al*.* reported a 12-week-flexibility-strength exercise with a focus on the upper and lower limbs resulted in a six-degree reduction in thoracic kyphosis [36]. Some studies have also investigated the effects of posture correction exercises on the elderly. For example, Sedaghati et al*.* (2019) assessed the effect of six weeks of selected corrective exercises (three times a week, 60 min each session) to correct kyphosis in elderly women and reported a significant reduction in the kyphosis angle [37]. The common point in these studies is the use of corrective exercises, focusing on correcting the back kyphotic position and forward-head posture. In addition to improving posture, corrective exercises can enhance respiratory function by relaxing pectoral muscle tension. Postural disorders, such as kyphosis, have been introduced as causes of lowered pulmonary function due to chest deformation. Similar studies have assessed the effects of respiratory or corrective exercises or even their combination of these exercises on the posture of patients with asthma. In this regard, Mirshafiei et al. (2021) investigated the effects of combined corrective and breathing exercises at home on the kyphosis angle and respiratory capacities of children with asthma and they declared significant differences in thoracic kyphosis curvature, and FVC/FEV1 ratio between the experimental and control groups [18], as well as significant improvements in the chest volume and expansion after the intervention [18]. Although these findings contradict our results regarding the chest anthropometric indicators, they might be caused by the differences between subjects enrolled in the two studies. Although our results agree with some studies mentioned, other studies have performed either breathing exercises (alone or in combination with physiotherapy) or corrective and strength exercises.

### Limitations

One of the limitations in this study was not comparing spirometry findings between COVID-19 patients and healthy individuals. Also, because of the coronavirus pandemic, access to a sufficient number of patients was difficult. However, we tried to select participants from both genders to diminish this problem. The lack of a follow-up procedure was the other limitation that could estimate the prolonged effects of the exercise program on patients. In addition, in the present study, like any other human research, the family environment and social and economic conditions of individuals can affect the results of the research. Therefore, the generalization of current results to the wider target population should be done cautiously.

## Conclusion

According to the present study's findings, the combination of corrective and breathing exercises could improve pulmonary function and correct cervical and thoracic posture in patients with a history of COVID-19 disease. Therefore, it seems that a comprehensive training program, including corrective and breathing exercises, can be helpful as a complementary treatment along with pharmaceutical therapy to reduce chronic pulmonary complications in patients infected with COVID-19.

## Supplementary Information

**Additional file 1.**

## Data Availability

All data generated or analyzed during this study are included in this published article.
